# Improving environmental exposure analysis using cumulative distribution functions and individual geocoding

**DOI:** 10.1186/1476-072X-5-23

**Published:** 2006-05-25

**Authors:** Paul A Zandbergen, Jayajit Chakraborty

**Affiliations:** 1 Department of Geography, University of South Florida, 4202 E. Fowler Ave, NES107, Tampa, FL 33620, USA

## Abstract

**Background:**

Assessments of environmental exposure and health risks that utilize Geographic Information Systems (GIS) often make simplifying assumptions when using: (a) one or more discrete buffer distances to define the spatial extent of impacted regions, and (b) aggregated demographic data at the level of census enumeration units to derive the characteristics of the potentially exposed population. A case-study of school children in Orange County, Florida, is used to demonstrate how these limitations can be overcome by the application of cumulative distribution functions (CDFs) and individual geocoded locations. Exposure potential for 159,923 school children was determined at the childrens' home residences and at school locations by determining the distance to the nearest gasoline station, stationary air pollution source, and industrial facility listed in the Toxic Release Inventory (TRI). Errors and biases introduced by the use of discrete buffer distances and data aggregation were examined.

**Results:**

The use of discrete buffers distances in proximity-based exposure analysis introduced substantial bias in terms of determining the potentially exposed population, and the results are strongly dependent on the choice of buffer distance(s). Comparisons of exposure potential between home and school locations indicated that different buffer distances yield different results and contradictory conclusions. The use of a CDF provided a much more meaningful representation and is not based on the *a-priori *assumption that any particular distance is more relevant than another. The use of individual geocoded locations also provided a more accurate characterization of the exposed population and allowed for more reliable comparisons among sub-groups. In the comparison of children's home residences and school locations, the use of data aggregated at the census block group and tract level introduced variability as well as bias, leading to incorrect conclusions as to whether exposure potential was higher at school or at home.

**Conclusion:**

The use of CDFs in distance-based environmental exposure assessment provides more robust results than the use of discrete buffer distances. Unless specific circumstances warrant the use of discrete buffer distances, their applcation should be discouraged in favor of CDFs. The use of aggregated data at the census tract or block group level introduces substantial bias in environmental exposure assessment, which can be reduced through individual geocoding. The use of aggregation should be minimized when individual-level data are available. Existing GIS analysis techniques are well suited to determine CDFs as well as reliably geocode large datasets, and computational issues do not present a barrier for their more widespread use in environmental exposure and risk assessment.

## Background

Geographic Information Systems (GIS) have been used extensively in recent years for the assessment of exposure to environmental pollution and related health risks. GIS technology is particularly well-suited for this research because it allows for the integration of multiple data sources (e.g., location of pollution sources and population characteristics), representation of geographic data in map form, and the application of various techniques (e.g., buffering) for proximity analysis [1-3]. Most GIS-based analyses of environmental risk follow a consistent outline. The first step is to identify the geographic boundaries of areas potentially exposed to pollution (impacted regions) based on the locations of facilities of concern in a study area. The next step is to estimate and compare the characteristics of the population within impacted regions with the characteristics of the population in other areas that are not exposed to pollution (comparison regions). Some studies have extended this general approach to examine the association between population characteristics and the magnitude of chronic pollution in impacted regions, measured by the frequency of toxic emissions, total quantity of emissions, or a ranking scheme that represents the degree of relative risk posed by each facility [4-6].

This overall approach, however, makes three major simplifications: 1) the use of one or more discrete distances in defining the spatial extent of potentially impacted regions; 2) the use of aggregated demographic data at the level of census enumeration units to derive the characteristics of the exposed population; and 3) the use of proximity as a surrogate for actual exposure conditions. Each of these factors is reviewed in some more detail.

### Use of discrete distances

Several approaches have been used in prior studies to define the spatial extent and shape of areas potentially exposed to environmental risks. The earliest and most basic approach uses pre-defined administrative boundaries or census enumeration units (e.g., ZIP codes, census tracts, or block groups) to define such areas [7-9]. The population at risk includes only those people who reside inside an enumeration unit hosting a polluting facility or point source. A key limitation of this "spatial coincidence" approach [[2] p. 20] is that edge effects are ignored. These effects are concerned with the possibility that a facility could be so close to the boundary of the host unit that a neighboring unit could be equally exposed to pollution. A resident in a census tract containing a toxic facility, for example, may live farther away from the facility than another person in an adjacent census tract which does not contain any facilities. Although the spatial coincidence approach facilitates statistical comparisons, it is based on the tenuous assumption that toxic pollution is restricted to the boundary of the spatial unit enclosing a facility.

An alternative approach for specifying the boundary of an impacted region consists of constructing a circular buffer at each potential pollution source. Several studies have used such buffers around facilities of concern to estimate areas and populations at risk [2,10-13]. Although a circular buffer provides a more realistic delineation of the area potentially exposed to pollution, there are two limitations associated with its application in environmental exposure analysis: (a) the radius of the circular buffer is chosen arbitrarily (e.g., as 1,000 yards, or one mile); and (b) buffers around all facilities in a study area typically have the same radius. The number, quantity, or toxicity of the substances stored or released at each individual facility are rarely incorporated in the construction of buffer zones [3]. Modifications to this approach include weighted exposures assigned to multiple buffer rings at increasing distances from the source.

These discrete GIS-based proximity assessments of environmental exposure are attractive due to their ease of calculation (one or more discrete buffer zones), their straightforward visual representation (circular rings around points sources), and the simplicity of the statistical tests required for determining significant differences (exposed vs. not-exposed populations). However, these discrete distances may be a poor surrogate for exposure potential, and do not reflect a continuous, more gradual reduction of exposure with increasing distance. Prior studies on environmental risk and exposure assessment have clearly indicated that the results are sensitive to the choice of buffer radius [2,12-15]. Using multiple buffers can overcome this limitation to some degree, but the determination of how many buffers to use and the choice of buffer radii remain ambiguous unless empirical values have been determined through field-based exposure monitoring, air dispersion modeling, or transport-fate exposure modeling. The chosen buffer radius or radii constrain(s) the spatial scale of the exposure analysis, and all results can be interpreted only within the context of this scale [16].

An alternative to the use of discrete distances is the use of continuous distances, i.e. determine the exact distance between all facilities of concern and the locations of the potentially exposed population. These distances can be described with a cumulative distribution function (CDF), which is a plot of the number of observations falling below every threshold value. Applied to the example of facilities of concern, a CDF would be plotted as distance versus potentially exposed population and would show how the size of this population (as a percentage of the total) increases with distance to the nearest facility. While CDFs have been used extensively in spatial analysis, they have not been adopted very widely in environmental risk and exposure assessment, despite the recognition they can overcome the limitations associated with the use of discrete distances [16]. CDFs can be particularly useful in making comparisons among sub-groups in the potentially exposed population [16,17]. This study therefore uses CDFs and illustrates how this methodology contributes to a better understanding of potential environmental exposure.

The application of CDFs to estimate the distance relationship of a population to facilities of concern, however, is not entirely new and has been utilized previously in the geographical literature. For example, [18] used a computer program to implement the CDF methodology before the advent of GIS technology; advances in automated spatial analysis techniques have made the development of distance-based CDFs more accessible and easier to apply.

While this study uses CDFs in combination with geocoded locations of potentially exposed individuals, this methodology can also be implemented with aggregated demographic data, in which case the centroid of the enumeration unit represents the population location. While not as detailed or accurate as the use of individual geocoded locations, the CDF approach still represents a potential improvement over the use of discrete distances when combined with aggregated demographic data. Examples of the application of CDFs to environmental exposure analysis using aggregated census data have started to appear in recent literature [19]. In such applications, however, CDFs are limited by the potential error and bias inherent in the use of aggregated demographic data, limiting their usefulness for analyzing sub-populations for which individual geocoded locations are not available.

### Use of aggregated demographic data

The second simplification in traditional environmental exposure analysis is the use of aggregated demographic data at the level of census enumeration units to derive the characteristics of the potentially exposed population. Environmental exposure analysis relies very heavily on demographic data collected by the US Census Bureau and other agencies, which are most commonly aggregated at the level of administrative boundaries or census units. It has been well documented that the choice of areal unit affects the comparability of studies and ultimately the strength and significance of statistical associations. This is known as the Modifiable Areal Unit Problem (MAUP) [20,21] and has been of some concern in environmental risk and environmental justice analysis [22-24]. One manifestation of the MAUP is the sensitivity of analytical results to the level of aggregation of the source data. This implies that different spatial units of analysis will produce different correlations, and in general the larger the unit of measurement, the stronger the correlation. These methodological issues have not been adequately addressed in the literature and remain a stumbling block for many types of analyses where ascribing aggregate data to all individuals who comprise the aggregate is inappropriate. In the context of proximity-based environmental exposure analysis, it creates problems for estimating the exposed population. For example, when using a 1-mile buffer around a facility of concern to determine the exposure population using census tracts, which tracts (or portions thereof) should be considered? For this type of polygon-on-polygon overlay analysis, a range of possibilities exists [13], including polygon containment (tracts which fall completely inside the buffer), centroid containment (tracts whose centroids fall inside the buffer), and simple areal interpolation (use the proportion of a tract's area that falls inside the buffer to determine the proportion of the population that resides inside the buffer, assuming uniform population distribution within each tract). All of these methods have been widely employed in environmental exposure analysis [2,11,25,26] and no single best technique has emerged. The application of dysametric mapping [27,28] in combination with areal interpolation has been suggested as a promising approach. Dysameteric mapping can be used to develop a more refined distribution of the population residing within a census enumeration unit using land cover information. However, dysametric mapping is somewhat cumbersome to carry out reliably, and is very sensitive to the assumptions about the population density difference among land cover categories. Despite these limitations, dysametric mapping represents a meaningful improvement on the use of the aggregated demographic data when geocoded locations of individuals or households are unavailable.

An alternative to the use of data aggregated at the level of census enumeration units is the use of individual street geocoded locations of the population of interest. This approach uses the addresses of individuals and a detailed street network to determine an accurate location for each individual or household. Street geocoding has become a relatively easy task in commercial GIS software and its application has seen a dramatic increased in recent years in many fields.

The key limitation for most health-related studies has logically been the availability of addresses of individuals. When individual health cases are reported (e.g., through doctor's visits, hospital visits, or community health screenings), access to these addresses are limited and, as a minimum, require a confidentiality agreement. When entire populations or sub-populations are used in environmental exposure analysis, individual addresses are usually unavailable and aggregated demographic data in the form of census enumeration units is the only alternative. Additionally, environmental exposure analysis is often concerned with rates, based on the number of instances within an appropriate universe of the population. While all instances can, in theory, be individually geocoded, the determination of rates requires some degree of aggregation. There are also some known biases in street geocoding, such as variable match rates and positional error [29-31]. These biases, however, are well documented and relatively minor for large areas; street geocoding is therefore expected to provide a more accurate distribution pattern of the potentially exposed population. Despite the difficulties of obtaining reliable and usable street address information, and some of the conceptual limitations associated with geocoding for population-level health analysis, an increasing number of health-related studies are employing street geocoding [39,33].

### The use of distance as a proxy for exposure conditions

The third simplification in traditional environmental exposure analysis is the use of proximity as a surrogate for actual exposure conditions. Underlying the use of proximity is the general assumption that there is a distance-decay function of the influence of the (potential) release. In reality, however, distance-decay functions will vary greatly depending on media of release (air, water, land), types of substance released (particulates, VOCs, etc) and local circumstances (e.g., wind, temperature, topography). Detailed information on the quantity and quality of pollution or emissions required to estimate such functions are often unavailable. Without the benefit of direct observation of exposure, however, distance has been very widely adopted as a proxy for exposure, i.e. exposure is some function of distance. The shape of the distance-decay function has been the subject of much debate [34,35]. Three common types include linear, square-root and logarithmic. Regardless of the shape of the underlying function, it needs to be emphasized that distance still remains a *proxy *for exposure; any analysis technique should be applicable to different types of functions and approaches that are not proximity-based.

Although distance to a pollution source is used to provide a preliminary estimate of potential exposure in this study, the actual extent and magnitude of exposure is not known and may not be a simple function of distance. Proximity is used to focus attention on how to summarize comparisons rather than assess the degree of exposure itself, as suggested by [17]. It should be noted that the use of CDFs in conjunction with individual geocoding does not in itself provide an accurate estimate of the degree of exposure, because it is still limited by the use of distance as a proxy. However, the general approach outlined in this paper is applicable to monitored or modeled exposure values; the use of distance as a surrogate therefore does not limit the evaluation of CDFs and individual geocoding in environmental exposure analysis.

### Research objectives and case-study

The objective of this study is twofold. The first objective is to demonstrate how cumulative distribution functions (CDFs) can be used to overcome the limitations associated with the use of discrete buffer distances in environmental exposure analysis. The second objective is to demonstrate how individual geocoding can be used to overcome the limitations associated with the use of aggregate demographic data in environmental exposure analysis.

To accomplish these two objectives a case study on school-aged children was conducted in Orange County, Florida. The case-study is used to answer the following general research question: Are school-aged children more likely to be exposed to environmental pollution and related health risks at home or at school? The limitations associated with the use of both discrete buffer distances and aggregate demographic data will be addressed using CDFs and individual geocoding.

Children were chosen as the subject of the case study since they represent the largest portion of the population that is susceptible to environmental health risks, and air pollution in particular [36,37]. The comparison between home and school locations for children in terms of exposure to environmental pollution and health risks reflect a long-standing interest to consider time-activity patterns in exposure assessment [38,39]. Time-activity databases such as the National Human Activity Pattern Survey (NHAPS) provide some insight into the locations to be considered when developing methodologies for exposure analysis. For example, according to the NHAPS data for the United States, youths from 11 to 17 years spend approximately 61% of their time indoors at home, 14% inside a school or other public building, followed by much smaller percentages for various other indoor and outdoor locations [40]. Many factors affect the exact nature of time-activity patterns [41], but several studies confirm that for children schools represent the second most important location (after the home) to consider in environmental exposure analysis [42-44].

The case-study of Orange County uses three different types of stationary environmental pollution sources: gasoline stations, small facilities with air releases from the EPA's Aerometric Information Retrieval System (AIRS), and large facilities with air releases from the EPA's Toxic Release Inventory (TRI). Gasoline stations and AIRS facilities represent relatively small and dispersed sources of air pollution, many of which are expected to be located in relatively close proximity to residential areas and schools. Gasoline stations are also likely to be located near major roadways and intersections. TRI facilities, on the other hand, represent larger sources of air pollution with some clustering in industrial areas, and most of these facilities are expected to be located at greater distances from residential areas and schools. The use of three different types of pollution sources thus provides an opportunity to explore the variability in the results and to reduce possible bias introduced by a unique spatial pattern of a single category of sources.

## Methods

### Locations of public schools and students

The locations of all public schools in Orange County were obtained from the Orange County Schoolboard. From this total set of 174 schools only the elementary, middle and high schools were selected, resulting in a total of 155 schools. Special schools were not included in the analysis, since detailed student enrollment records for these schools were not available; the overall enrollment in these 19 special schools is relatively small: 6,858 students out of a total of 173,334 students in the public school system in Orange County in 2005.

Student enrollment records for 2005 were obtained from the Orange County Schoolboard for all public elementary, middle and high schools in Orange County. The home residences of these students were street geocoded using StreetMap USA for ArcGIS 9. Of a total of 163,886 records, 155,923 records (95.1%) could be reliably geocoded. Student records that did not allow for a determination of the school they were attending were removed from the sample, resulting in a final set of 151,709 geocoded students' home residences, with reliable information on which school they were attending. It should be noted that the total sample of home residences includes many duplicates, reflecting siblings living at the same physical address. Duplicate locations were maintained since they represent different students.

While enrollment totals were available for each school, the geocoded student records were used in determining the enrollment total for each school to avoid potential biases in geocoding match rate across the study area. Therefore, the combined enrollment for all schools considered was also 151,709. This allows for a direct comparison of the student population at home and at school.

**Figure 1:**
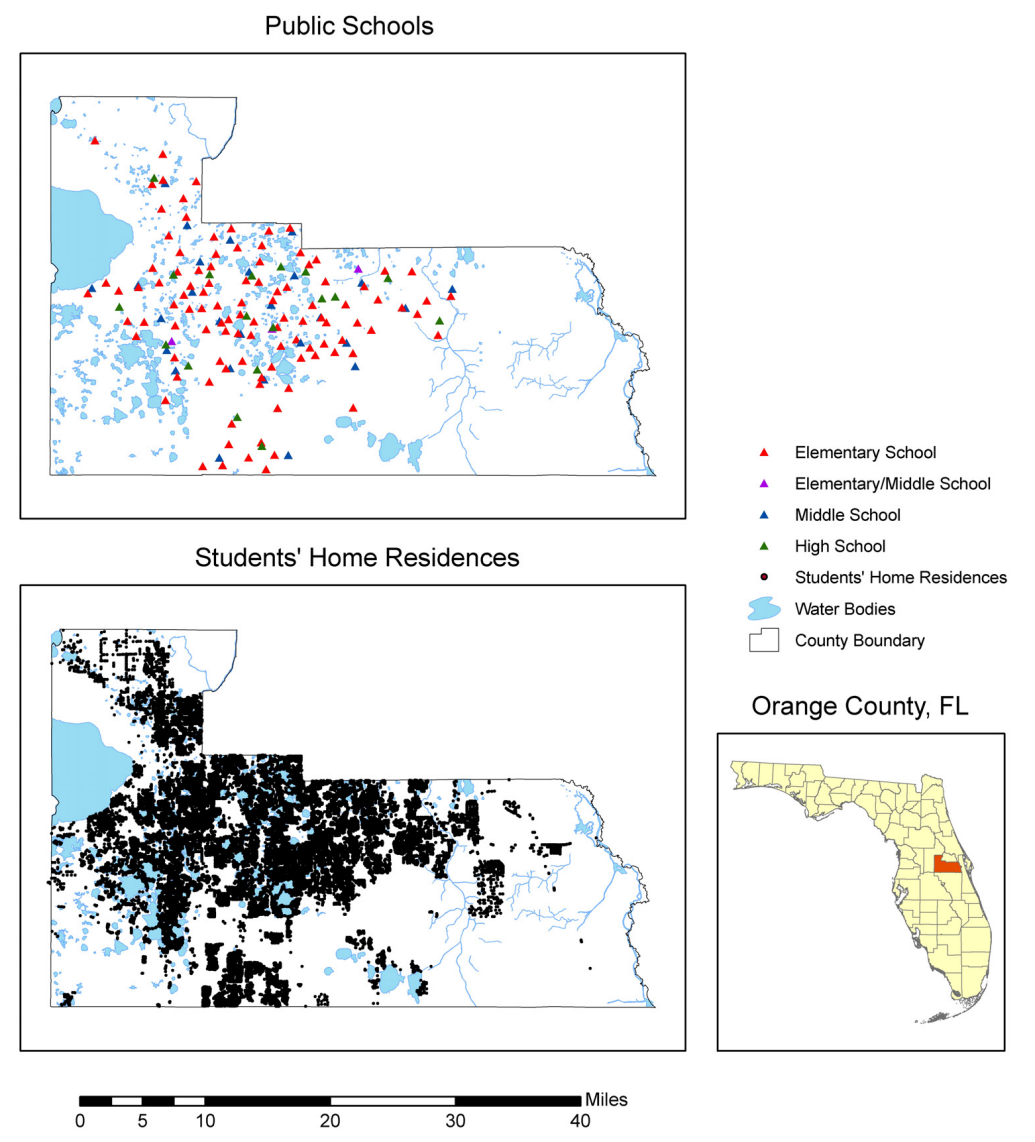
Location of public schools and home residences of students attending public schools in Orange County, Florida in 2005.

Figure 1 shows the location of all public elementary, middle, and high schools in Orange County, as well as the locations of all the street geocoded students' home residences.

### Locations of potential sources

The three types of pollution sources analyzed in this case study include gasoline stations, small and stationary sources of air pollution, and industrial facilities listed in the Environmental Protection Agency (EPA)'s Toxic Release Inventory. These are described in more detail below.

#### Gasoline stations

The georeferenced locations of all gasoline stations in Florida were obtained from the Florida Geographic Data Library (FGDL). The original data was obtained from the online Yellow Pages and Super Pages in 2004, and the locations were geocoded using ARC Logistics Route based on GDT Roads. The geocoding match rate is not known. A total of 3,456 georefenced gasoline stations were found in the data provided by FGDL.

#### AIRS facilities

Facilities with air releases were obtained from the EPA's Aerometric Information Retrieval System (AIRS). This database contains mostly small facilities that do not fall under the Toxic Release Inventory reporting requirements. Examples of facilities in the AIRS include drycleaning facilities, auto repair shops, and small manufacturing facilities. Due to the large number of records in this database, only the facilities in Orange County and adjacent counties were obtained (including Seminole, Osceloa, Polk, Lake, Volusia and Breward Counties). A total of 670 facilities were identified. 158 of those had reliable latitude/longitude information and could be mapped directly. The remaining 511 facilities were street geocoded using StreetMap USA for ArcGIS 9. A total of 407 facilities could be reliably geocoded resulting in a geocoding match rate of 79.6%. Combining the facilities mapped using latitude/longitude fields and the street geocoded facilities resulted in a total of 565 facilities, or 84.3% of all facilities in the 7 Counties.

#### TRI sites

Data on facilities in EPA's Toxic Release Inventory (TRI) were obtained from the TRI database (2000–2003). All facilities in Florida reporting to the TRI were identified without considering the nature of the chemicals in the inventory. Since the TRI database contains a wide variety of release types (air, land, water) and release quantities can vary substantially from year to year, only those facilities with a combined stack air release of at least 1,000 pounds for a four-year period (2000–2003) were selected for further analysis. A total of 317 facilities for Florida were identified. 190 of those had reliable latitude/longitude information and could be mapped directly. The remaining 127 facilities were street geocoded using StreetMap USA for ArcGIS 9.0. 89 facilities could be reliably geocoded resulting in a geocoding match rate of 70.0%. Combining the facilities mapped using latitude/longitude fields and the street geocoded facilities resulted in a total of 279 facilities, or 88.0% of all facilities in all of Florida. Of the 21 sites recorded for Orange County, 21 could be reliably located.

**Figure 2:**
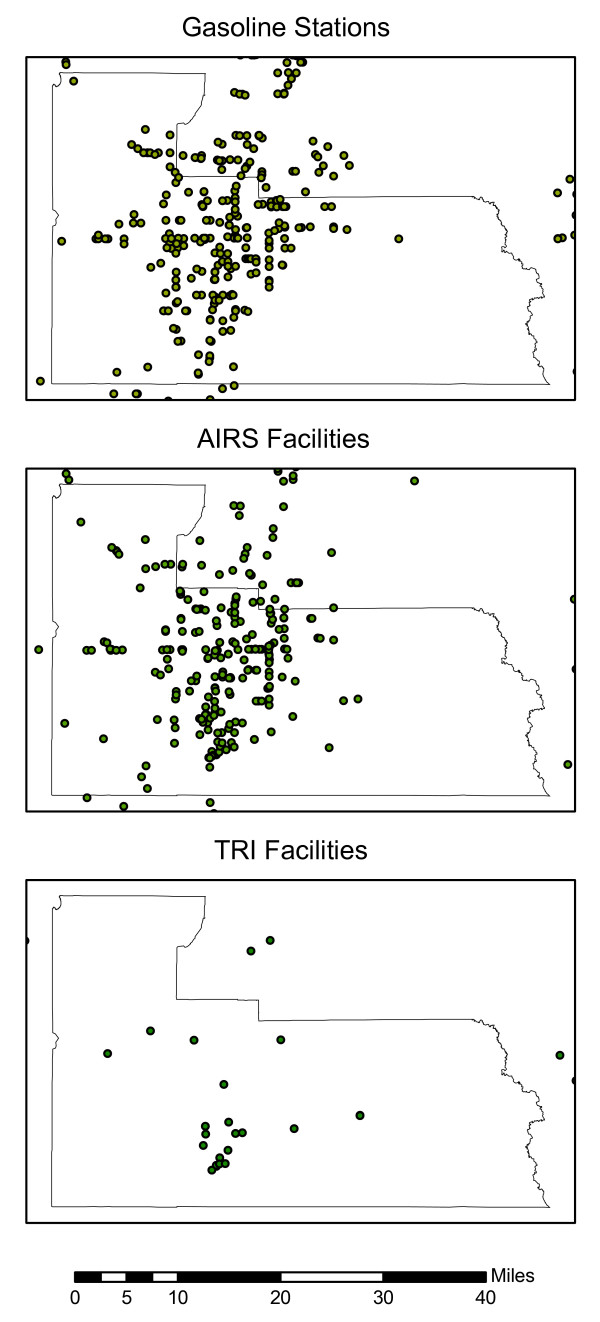
Location of potential point-sources of air pollution: gasoline stations, AIRS facilities and TRI sites in Orange County, Florida in 2005.

Figure 2 shows the locations of all the geocoded locations of gasoline stations, AIRS facilities and TRI sites in Orange County and surrounding areas.

### Census data

Boundaries of census tracts and block groups for 2000 were obtained from the Florida Geographic Data Library (FGDL) for all of Florida. These original boundaries were modified using a detailed hydrography layer for Orange County. Due to presence of a large number of surface water bodies (some of substantial size) dasymetric mapping [27,28] was deemed necessary in order not to overestimate the surface area of census enumeration areas. Since none of the available hydrography corrected surface area estimates for census tracts and block groups where deemed sufficiently reliable, the surface area was calculated by subtracting the areas of all surface waterbodies as derived from a detailed hydrography layer for Orange County. Centroids were determined for every tract and block group using the hydrography-corrected polygons, but centroids were allowed to fall inside waterbodies. The number of students residing in each census tract and block group was determined through a spatial overlay analysis between the census polygons and geocoded students' home residences.

### Distance analysis

All spatial data was re-projected in the appropriate State Plane coordinate system for Orange County with units in US Survey Feet, as this was deemed the most accurate coordinate system for distance calculations within the study area. Distance to the nearest facility of each type (gasoline stations, AIRS facilities and TRI sites) was determined for schools, students' home residences, tract centroids and block group centroids. Distance values were rounded to the nearest feet. Cumulative distribution functions (CDFs) were created for each of the three potential pollution sources, plotting children as a % of the total number versus distance to nearest facility in feet. Schools, tracts centroids and block group centroids were weighted with the number of children associated with each, which allows for a direct comparison of each of the CDFs for a single type of facility. To simulate the effect of using discrete distances commonly used in GIS-based buffer analysis, the cumulative number of students was also determined using distances of 0.25, 0.5, 1, 2 and 4 miles. These discrete values represent the most widely used buffer radii reported in proximity-based environmental exposure analyses [11-15]. While obtained from the results of the continuous distance analysis, they simulate the results of using discrete uniform buffers of these sizes.

### Statistical analysis

The CDFs were compared using a Kolmogorov-Smirnov (K-S) two-sample test. The K-S two sample test is based on the maximum absolute difference (D) between the CDFs for two continuous random variables. Unlike conventional statistical tests, this is a non-parametric test that does not require the variables to be normally distributed. The null hypothesis for the K-S test assumes there is no different in the CDFs associated with the two groups (i.e. school and home locations). For all comparisons reported in this paper, the largest observed difference between the two CDFs being examined was compared to the critical value of D at the 5 percent level of significance to determine if there is a statistically significant difference between the curves.

The percentages of children at home and at school for various buffer distances were compared using a two-sample Z-test of proportions. The null hypothesis for this parametric test based on independent samples assumes that the proportions of students in each group are equal.

## Results and discussion

**Figure 3:**
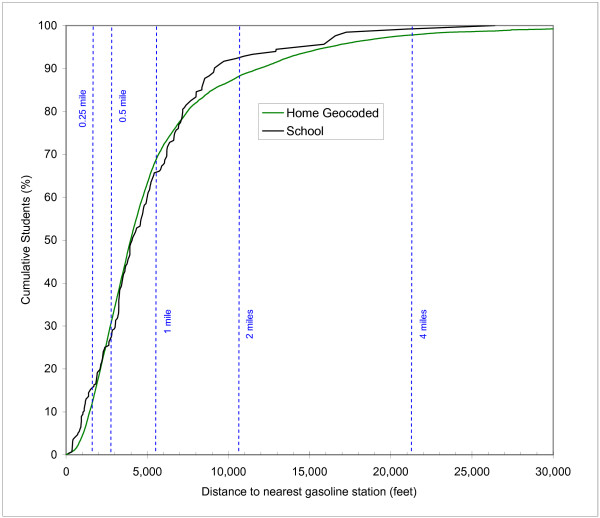
Cumulative Distribution Function of distance to nearest gasoline station of students at school versus geocoded home locations.

Figure 3 shows an example CDF of the distance to the nearest gasoline stations for the student population at school and at home (based on geocoding). This figure is used to explain the use of the CDF and to highlight some important characteristics. The Y-axis shows the cumulative number of students as a % of the total (151,709). Logically, as the distance from facilities of concern increases, so does the percentage of the student population. At a distance of zero, the percentage of students is (almost) zero, and the percentage reaches a value of 100% at a very large distance of more than 5 miles. Two different curves are shown: one for the student population at school and one for the student population at home based on geocoding. In creating the curve for the student population at school, each school was weighted by the number of students associated with that school. A closer look at the curve for school locations reveals a somewhat step-wise pattern: since there are 155 schools, there are in fact 155 unique steps in the curve, with the step height determined by the student enrollment at that school. It is also worth noting what the meaning is of one curve being higher than the other at a particular distance. For example, if the curve for school locations is higher than the curve for home locations at a particular distance, it means that the percentage of students that resides within that distance of the nearest gasoline station is larger for school locations than for home locations. When using distance as a proxy for exposure, at that distance students are potentially more exposed at school than at home. Following previous research that has utilized distance as a proxy for environmental exposure [14,17], the term "exposure potential" is used in the subsequent discussion of the results to indicate the percentage of the total number of children potentially exposed at home compared to at school, and is plotted on the Y-axis of the CDF curves.

Figure 3 indicates that the determination of the highest curve varies with the distance being considered: at distances up to 2,500 feet the curve for schools is higher, between 2,500 and 7,000 feet the curve for home locations is higher, and above 7,000 feet the curve for schools is higher again. This clearly indicates that determining whether students are more exposed at school or at home strongly depends on the distance considered. The discrete distances of 0.25, 0.5, 1, 2 and 4 miles are also plotted in Figure 3 and the numerical results associated with these distances summarized in Table 1. The limitations of conventional buffer analysis based on discrete distance values can be assessed from this table. A buffer analysis using a 0.25 mile radius would indicate that 13.02% of students at school locations and 8.20% of students at home reside within the buffer, suggesting exposure potential at school is of greater concern. A buffer analysis based on a 0.5 mile radius would indicate that 26.37% of students at school locations and 28.63% of students at home reside within the buffer, suggesting exposure potential at home is of greater concern. The results continue to vary with larger buffer distances: higher percentages for schools at 0.25, 2 and 4 miles, and lower percentages at 0.5 and 1 mile. It should be recognized that discrete buffer distances are normally chosen without any knowledge of the actual empirical CDF; based on the results in Figure 3, this is very likely to result in an inaccurate and incomplete characterization of the exposure potential, and will lead to biased results, in particular since the difference at any arbitrarily chosen distance will often be statistically significant.

**Table 1:** Comparison of student population at school to student population at home (geocoded) locations using discrete buffer distances from gasoline stations.

Distance		School locations		Geocoded home locations		% Difference
Miles	Feet	No. of students	% of students	No. of students	% of students	
0.25	1320	19,755	13.02%	12,444	8.20%	4.82%
0.50	2640	40,009	26.37%	43,434	28.63%	-2.26%
1.00	5280	96,262	63.45%	100,695	66.37%	-2.92%
2.00	10560	140,064	92.32%	133,519	88.01%	4.31%
4.00	21120	150,466	99.18%	148,306	97.76%	1.42%

	Total	151,709	100.00%	151,709	100.00%	

**Table 2:** Largest absolute difference (Kolmogorov-Smirnov D) from cumulative distribution function for school locations. All differences significant at .05 level, based on critical value of 0.00349

Cumulative distribution function	Gasoline stations	AIRS facilities	TRI facilities
Geocoded home locations	0.05352	0.01217	0.04091
Tract centroid	0.08856	0.04965	0.05043
Block group centroid	0.07389	0.07360	0.05222

In the remaining CDFs, the distance along the X-axis is plotted on a logarithmic scale to assists in the visual interpretation of the curves at the lower distances; exposure potential is assumed to decrease with increasing distance, and the lower range of the distance is therefore of most interest and not very easy to interpret using a linear scale.

**Figure 4:**
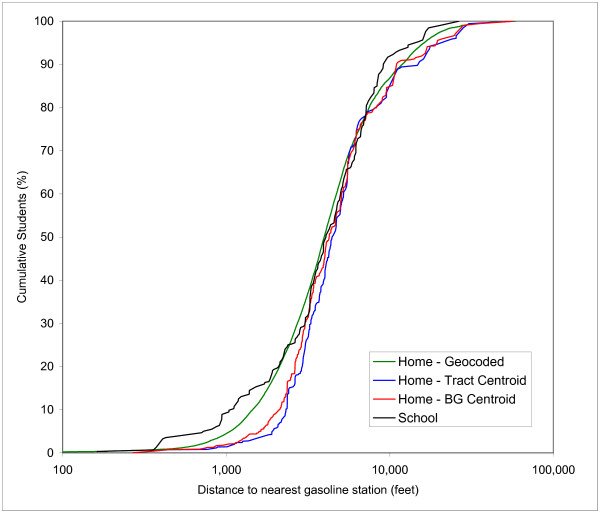
Cumulative Distribution Function of distance to nearest gasoline station of students at school versus three estimates of home locations.

**Figure 5:**
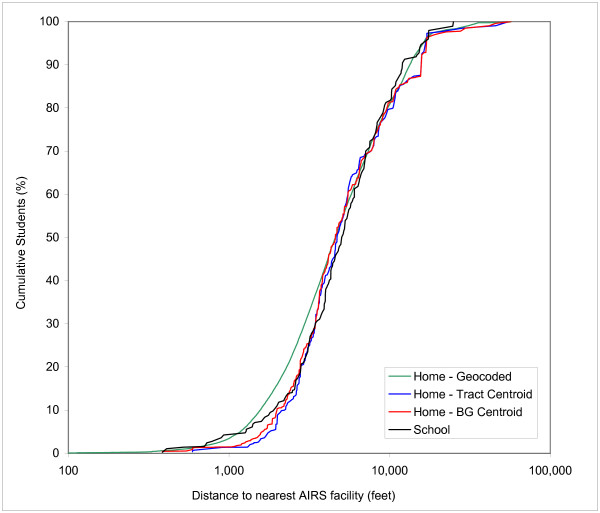
Cumulative Distribution Function of distance to nearest AIRS facility of students at school versus three estimates of home locations.

**Figure 6:**
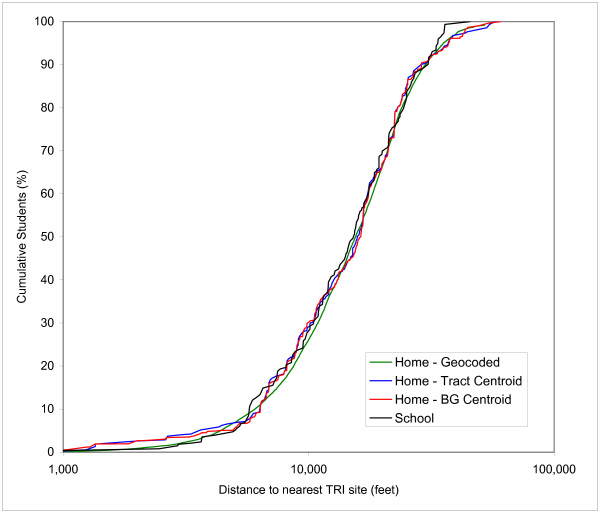
Cumulative Distribution Function of distance to nearest TRI sites of students at school versus three estimates of home locations.

Figures 4, 5, and 6 show the CDFs for gasoline stations, AIRS facilities and TRI sites, respectively. In each Figure four curves are depicted: one for schools and three for the students' home residences. These three versions represent the street geocoded home locations, the block group centroids and the tract centroids. Each Figure therefore shows 3 different home/school comparisons. The curve for the geocoded home locations represents the "true" distribution, while the block group and tract centroids represent the effect of aggregation at the level of census enumeration units. The discrete buffer distances are not shown in Figures 4, 5 and 6, but the numerical results for the percentages at these distances are summarized in Table 3.

**Table 3:** Z-Test results for discrete buffer analysis. Results labeled with * are significant using p < 0.01.

Buffer radius (miles)	0.25	0.5	1	2	4
Gasoline stations:					

Geocoded home locations:					

School	13.02%	26.37%	63.45%	92.32%	99.18%
Home	8.20%	28.63%	66.37%	88.01%	97.76%
Difference	4.82%*	-2.26%*	-2.92%*	4.31%*	1.42%*

Tract centroid:					

School	13.02%	26.37%	63.45%	92.32%	99.18%
Home	2.71%	17.57%	60.56%	83.56%	89.78%
Difference	10.31%*	8.80%*	2.90%*	8.76%*	9.41%*

BG centroid:					

School	13.02%	26.37%	63.45%	92.32%	99.18%
Home	3.63%	20.97%	61.57%	85.14%	95.92%
Difference	9.39%*	5.40%*	1.88%*	7.18%*	3.26%*

AIRS facilities:					

Geocoded home locations:					

School	5.58%	16.87%	53.44%	84.36%	97.95%
Home	6.76%	25.20%	56.30%	82.57%	97.86%
Difference	-1.18%*	-8.33%*	-2.86%*	1.79%*	0.08%

Tract centroid:					

School	5.58%	16.87%	53.44%	84.36%	97.95%
Home	1.44%	14.45%	57.04%	80.28%	97.60%
Difference	4.14%*	2.42%*	-3.60%*	4.08%*	0.34%*

BG centroid:					

School	5.58%	16.87%	53.44%	84.36%	97.95%
Home	2.88%	17.80%	57.16%	81.46%	97.23%
Difference	2.70%*	-0.93%*	-3.72%*	2.90%*	0.72%*

TRI facilities:					

Geocoded home locations:					

School	0.18%	0.79%	6.01%	30.29%	70.73%
Home	0.34%	1.55%	7.78%	28.52%	70.71%
Difference	-0.16%*	-0.76%*	-1.78%*	1.77%*	0.03%

Tract centroid:					

School	0.18%	0.79%	6.01%	30.29%	70.73%
Home	0.55%	3.23%	6.95%	30.54%	70.09%
Difference	-0.37%*	-2.44%*	-0.95%*	-0.25%	0.64%*

BG centroid:					

School	0.18%	0.79%	6.01%	30.29%	70.73%
Home	1.20%	3.40%	6.69%	31.32%	69.72%
Difference	-1.02%*	-2.62%*	-0.69%*	-1.03%*	1.02%

The first general observation regarding the CDFs is that the curves for block groups and tract centroids display a stepwise pattern similar to the curves for the schools, reflecting the fact that the data is aggregated for 369 block groups and 193 census tracts. The second general observation is that the curves for gasoline stations and AIRS facilities are more similar to each other than those for TRI sites. For example, the 50^th ^percentiles for street geocoded students' home residences are 3,970 and 4,595 feet for gasoline stations and AIRS facilities, respectively, while for TRI sites the value is 15,518 feet. This is a reflection of the fact that there are many more gasoline stations and AIRS facilities than TRI sites, and they are also more spatially dispersed, as evidenced in Figure 1. This results in a relatively large proportion of students living in close proximity to these two types of facilities.

In the following, the results for each type of facility will be explored in more detail. Table 2 shows the results of the Kolmogorov-Smirnov test for the CDFs in Figure 4, 5 and 6. The distribution of the exposure potential at schools locations is compared to the three different distribution for the students' home residences. This is repeated for the three different types of facilities, for a total of nine K-S tests. Table 2 reports the D_max _values, which represent the largest difference between two distribution on a scale of 0 to 1. All differences were found to be significant at the 0.05 level. The K-S test results indicate that the exposure potential between school and home locations is statistically different for each of the three versions of home location being considered; however, it does not provide any insight into the specific distance ranges where this difference is most significant or even what the sign of the difference is.

Figure 4 shows the CDFs for proximity to gasoline stations. Comparing the curves for school locations and geocoded home residences reveals that exposure potential at school is higher from 0 to 2,500 feet, lower from 2,500 to 7,000 feet and higher again at distance greater than 7,000 feet. This pattern does follow a certain logic related to the nature of gasoline stations: most gasoline stations are found on major intersections and along major roads. There are very few, if any, that are located immediately adjacent to a residential area, but it seems likely that at least some of them are located in close proximity to a few schools. This is shown by the relatively large difference in the 500 to 2,000 feet distance range in Figure 3. The very large distances are mostly associated with the rural areas within the County; students' home residences tend to be dispersed, but schools in these rural areas are likely to be in close proximity to a major road or within a more densely developed part of the rural areas, and therefore in closer proximity to other urban facilities, including gasoline stations. This is shown by the relatively large difference in the 7,000 to 12,000 feet distance range. The curves for the census block group and tract centroids indicate an interesting pattern: these curves are consistently lower than the control curve that represents geocoded home locations. This is particularly pronounced at lower distances. For example, the 10^th ^percentile for geocoded home locations is 1,450 feet, while for block group and tract centroids the values are 2,120 and 2,321 feet, respectively. This is a direct result of the use of polygon centroids: gasoline stations tend to be located along major roads which coincide with some of the boundaries of census enumeration areas. Consequently, the use of block group and tract centroids underestimates exposure potential in the short distance range. And while the control curve for geocoded home residences exceeds the curve for schools location for a substantial distance range, this only happens sporadically for the curves for block groups and tract centroids. It can also be noted that the underestimation of exposure potential is more pronounced for the tract centroids than for block group centroids as a result of the higher degree of aggregation (193 tracts versus 369 block groups in Orange County).

Figure 5 shows the CDFs for proximity to AIRS facilities. A comparison of the curves for school locations and geocoded home residences reveals that exposure potential is nearly identical up to 1,000 feet and then shows a much higher value for geocoded home locations from 1,000 to 8,000 feet, above which the curves are much closer again. The hypothesis behind this pattern in exposure potential is that some of the AIRS facilities are located in light industrial areas, some of which are directly adjacent to residential areas, in particular in the urban-rural fringe zone. A closer look at the pattern in Figure 1 reveals that while AIRS facilities display some of the same clustering as gasoline stations, their pattern is slightly more dispersed, with more of them located outside the most densely urbanized areas of Orange County. Looking at the curves for the block group and tract centroids, an underestimation of the exposure potential similar to the pattern observed for gasoline stations occurs, with the two curves falling below the control curve that represents geocoded home locations. Not only does the use of block group and tract centroids underestimate the exposure potential for much of the distance range considered, it also produces estimates that are lower than for the school locations for distances up to approximately 2,500 feet.

Figure 6 shows the CDFs for proximity to TRI sites. Compared to the other two facilities considered, the curves are much closer together. While the K-S tests confirm the differences are statistically significant, the absolute values of the difference are much lower across most of the distance range. This is not strongly reflected in the D_max _values, however, as a result of a substantial "spike" in the distribution for the school locations around 35,000 feet. The hypothesis for the similarity of the curves is the fact that there are fewer TRI sites, resulting in larger distances overall as already mentioned before. Comparing the curves for school locations and geocoded home residences reveals that exposure potential is very similar up to approximately 5,500 feet and then shows a higher value for school locations at larger distances. Looking at the curves for the block group and tract centroids, a pattern opposite to that of gasoline stations and AIRS facilities occurs, i.e. an overestimation of the exposure potential, with the two curves falling above the control curve of geocoded home locations. This is particularly strong in the lower distance ranges up to 4,000 feet, which are of most concern for environmental exposure analysis.

Moving on from the CDFs, Table 3 shows the Z-test results for the discrete buffer analysis, including 0.25, 0.5, 1, 2 and 4 miles. At each distance, a comparison is made between the percentage of students inside the buffer zone based on school and home locations; the Z-test is based on the difference of the proportions, using the total student population as the sample size (151,709). Three different comparisons are made for each of the three representations of the home locations. The 3 comparisons at 5 distances are repeated for each of the 3 types of facilities, resulting in a total of 45 Z-tests. Out of this total of 45 comparisons, 41 were found to be statistically significant at the 0.01 level.

The results for gasoline stations reveal several interesting patterns. For the comparison between school locations and geocoded home residences, the direction of the difference changes twice with increasing buffer size. For a buffer of 0.25 miles, exposure potential is higher at schools (+4.82%); for buffers of 0.5 and 1 mile exposure potential is higher at home (-2.26% and -2.92%, respectively); and for buffers of 2 and 4 miles exposure is higher again at schools (+4.31% and +1.42%, respectively). While this general pattern was suggested previously by Figure 4, the discrete distance analysis reveals the numerical values of these differences and more importantly confirms that the difference is statistically significant at every buffer distance considered. This clearly illustrates the limitations of the use of discrete buffer zones to characterize exposure potential. Results for block group and tract centroids reveal higher exposure potential at schools at all buffer distances considered, resulting from the underestimation of exposure potential discussed above for the CDFs.

The results for AIRS facilities show a somewhat different pattern but also confirm the sensitivity of the analysis results to the chosen buffer distance. For buffers of 0.25, 0.5, and 1 mile, potential exposure at the geocoded home locations is higher than at school locations, but not at 2 and 4 miles. For block groups and tracts centroids, the higher exposure at home locations is only observed at buffer distances of 0.5 and 1 mile, and 1 mile, respectively.

The results for TRI sites show yet another pattern. Values for the difference in proportions in general are much lower than for the other two types of facilities, and three of the nine Z-tests were not statistically significant at the 0.01 level. The comparison of school locations and geocoded home locations reveals that exposure potential is higher at home for buffer distances of 0.25, 0.5 and 1 mile but higher at school for a buffer distance of 2 miles. Census block groups and tract centroids reveal a fairly similar pattern, with exposure potential higher at home at all buffer distances with the exception of 4 miles.

## Conclusion

This study has demonstrated the application of cumulative distribution functions (CDFs) to overcome the limitations of discrete buffer distances commonly used in the assessment of environmental exposure and related health risks. The use of discrete buffers distances in proximity-based exposure analysis can introduce substantial bias in terms of determining the potentially exposed population, since the results are strongly dependent on the chosen buffer distance(s). The results of this study have emphasized the limited reliability of the use of a single discrete buffer distance. The comparison of exposure potential between home and school locations, based on tests for statistical significance, indicated that different buffer distances yield different results and contradictory conclusions. If, and at what distance, this change occurs cannot be known *a-priori *since it requires knowledge of the CDF. The selection of any particular discrete buffer distance, therefore, is not justifiable unless there is some other clearly established basis for choosing a particular value, such as regulatory requirements or empirically derived estimates of meaningful distances using exposure monitoring. The use of a continuous distance function provides a much more meaningful representation, and does not *a-priori *assume that any particular distance is more relevant than another. As a minimum, proximity-based exposure analysis should employ the use of multiple discrete buffer distances, as well as determine the sensitivity of the buffer analysis result to the chosen distance values.

The use of the CDFs was demonstrated using distance as a proxy for exposure, which may have very little correlation with the actual doses of the released chemicals received by an individual. However, the technique is applicable to any type of spatially explicit exposure characterization, derived through fate-transport modeling or field-based exposure monitoring.

This study has also demonstrated the utilization of individual geocoded locations to overcome the limitations of aggregated demographic data in environmental exposure analysis. The use of individual geocoded locations provides a more accurate characterization of the exposed population and more reliable comparisons among sub-groups. In the comparison of school and home residence locations of school children, the use of data aggregated at the census block group and tract level introduced variability as well as bias, leading to incorrect conclusions as to whether exposure potential was higher at school or at home. The specific results presented here are sensitive to the chosen overlay technique (centroid containment of census enumeration areas); however, the objective of this study was not to compare differences in overlay techniques, but to demonstrate how aggregation introduces analytical bias using individual geocoded locations as a control.

The results also indicate that the effect of spatial aggregation will vary with the nature of the distance relationship considered. As expected, the effect of aggregation declines at larger distances. Again, the exact nature of the bias introduced by aggregation cannot be predicted *a-priori*, and therefore the CDF provides a meaningful way to determine the magnitude and scale extent of the variability and bias resulting from aggregation.

While the use of CDFs in combination with individual geocoding for environmental exposure analysis has demonstrated potential, a number of limitations need to be recognized. First, addresses for individuals may not be accessible and complete addresses for an entire population or sub-populations are typically unavailable. There are also some known errors and potential biases in street address geocoding. In addition, many health studies require the estimation of rates instead of instances, which necessitates some degree of aggregation for comparative purposes. CDFs are most powerful when applied to individual geocoded locations, but they can be used with aggregated demographic data (e.g., using the centroid of the census enumeration unit). Applications involving aggregated data, however, introduce some potential bias, limiting the utility of CDFs for fine-scale analysis when individual geocoded locations cannot be used.

The comparison of school and home locations in terms of exposure potential from three different pollution sources revealed different patterns for each source. Under the (preliminary) assumption that the shortest distances (within several thousand feet) are of greatest concern, exposure potential to gasoline stations is higher at school and exposure potential to AIRS facilities and TRI sites is higher at home. In terms of total exposed population of children within these shortest distances, gasoline stations are of highest concern, followed by AIRS facilities and TRI sites. These findings do not reflect the relative amount of time spent at home and at school, nor do they reflect the different nature of the exposure potential from various sources. The results of this case study, however, do follow a consistent methodology for characterizing proximity-based environmental exposure analysis which can be extended to any type of spatially explicit exposure model and is thus flexible enough to be applicable in multiple contexts and situations.

While the present study considered all school-aged children attending public schools as a single population, future research will utilize CDFs and individual geocoding to investigate racial/ethnic and socio-economic disparities in exposure potential within the study area.

## Competing interests

The author(s) declare that they have no competing interests.

## Authors' contributions

PAZ obtained and processed the data for schools, student records, gasoline stations, AIRS facilities and census data. JC obtained and processed the data for TRI facilities. PAZ performed all the street geocoding, as well as the distance analysis. JC performed the statistical testing.
